# Survival of immediately versus delayed loaded short implants: A prospective case series study

**DOI:** 10.4317/medoral.20407

**Published:** 2015-06-02

**Authors:** Joaquin Alvira-González, Erick Díaz-Campos, Maria-Angeles Sánchez-Garcés, Cosme Gay-Escoda

**Affiliations:** 1 DDS, Master Degree Program in Oral Surgery and Implantology, University of Barcelona (Spain); 2 DDS, Fellow of Oral Surgery and Implantology, School of Dentistry, University of Barcelona (Spain); 3 MD, DDS, MS, PhD. Associate Professor of Oral Surgery, Proffesor of Master Degree Program in Oral Surgery and Implanto-logy, Faculty of Dentistry - University of Barcelona, (Spain). Researcher of the IDIBELL Institute, Barcelona (Spain); 4 MD, DDS, MS, PhD, EBOS. Chairman and Professor of Oral and Maxillofacial Surgery, Faculty of Dentistry, University of Barcelona. Director of Master’s Degree Program in Oral Surgery and Implantology (EHFRE International University/FUCSO). Coordinator/Researcher of the IDIBELL Institute. Head of Oral and Maxillofacial Surgery Department of the Teknon Medical Center, Barcelona (Spain)

## Abstract

**Background:**

To assess and compare survival rates of immediately and delayed loaded short implants (7 mm) in free ends of a partially edentulous jaw with moderate-severe alveolar bone resorption.

**Material and Methods:**

24 patients with atrophic edentulous free-ends were included in this prospective study. Four study groups were monitored monthly and their behavior was evaluated: bridges supported only by short implants and mixed short and long implant bridge groups, both with immediate and delayed loading. Failures, bone loss, probing depth and bleeding on probing were evaluated.

**Results:**

54 Mk III Shorty TiU and 15 Brånemark System®MK III TiU implants with a length longer than 7mm were included in the study. Twenty-eight implants were inserted following the immediate loading protocol and 26 according a two-stage procedure, depending on the torque value. The cumulative survival rate of short implants was 87% (n=54) after a mean time of 47.72 months (range 33-62 months), showing statistically significant differences related to loading protocol (*p*=0.047). Short implants immediately loaded had a higher long-term survival rate (96.4%) compared to the other study group (76.9%). Besides, short implants splinted to longer immediately loaded implants presented the highest survival rate (100%). Twenty-five (53.19%) short implants showed a bone loss of less than one millimeter after the follow-up period. Statistically significant differences were found between bleeding on probing, presence of plaque or suppuration and a higher bone loss in both loading protocols (*p*=0.001).

**Conclusions:**

Immediate loading of short implants placed on free ends can be considered an option in the treatment protocol of patients with severe bone resorption especially if implants are splinted to others of greater length.

**Key words:** Dental implants, short implants, immediate loading, prospective study, TiUnite surface.

## Introduction

There are many treatment options to restore posterior regions of a partially edentulous atrophic maxilla and mandible. The degree of resorption and the proximity of anatomical structures (maxillary sinus and inferior alveolar nerve) has, in many cases, an influence on the treatment planning, which ranges from major surgery techniques (extraoral autologous grafts, osteogenic bone distraction, mandibular nerve transposition) to minor surgical procedures (split crest, tilted implants, guided bone regeneration techniques, mono cortical block grafts, elevation of the sinus floor membrane and short implants) (1-3).

Patients today demand techniques as atraumatic as possible, and with an early/immediate loading. It is then necessary to find faster and less invasive procedures (4).

The definition of ‘‘short’’ implants is controversial because some authors consider as ‘‘short’’ all implants with a length within the range of 7–10mm, whereas other authors consider as ‘‘short’’ those implants of 8mm or less (5,6). Nevertheless, it is commonly perceived that implants 7mm or shorter do not have a good long-term prognosis when compared with their longer counterparts (6). However, current data suggest that the same level of clinical success may be reached for short implants compared to longer implant. In fact, survival rates from 88% to 100% have been reported (3,7,8).

The primary outcome of this study is to compare the survival rates of immediate loaded short implants versus short implants that were loaded following a delayed protocol in posterior areas of partially edentulous jaws with moderate-severe alveolar bone resorption. Secondary, the authors want to present preliminary findings on survival rates of immediate loaded short implants, considering short implant bridge (prosthesis supported only by short implants) or mixed bridge (short implants splinted to longer implants). The main hypothesis was that survival rate of short implants following an immediate loading protocol and splinted to a longer implants is similar to survival rates of conventionally loaded short implants described in the literature.

## Material and Methods

A total of 26 patients were treated and followed up from January 2007 until June 2012. The study was approved by the Research Ethics Committee (CEIC) of the Dental Clinic of the University of Barcelona. Before enrollment, all patients received explanations regarding the objectives, implications and possible complications of this study and agreed to participate by signing an informed consent.

The inclusion criteria of this prospective case series study were as follows: patients over 18 years, without uncontrolled systemic diseases, who had not been previously treated with radiotherapy or with guided bone regeneration prior to, or simultaneously to, implant surgery and with at least one upper or lower edentulous free end and with no possibility, in one or more of the implant locations planed, to insert an implant ≥ 8.5 mm without risk to damage anatomical structures. Exclusion criteria consists of the following: Medical and/or general contraindications for surgical procedures (ASA score ≥ III), presence of active clinical periodontal disease in the dentition determined by probing pocket depths of ≥5 mm and bleeding on probing or suppuration and heavy smoking habit (≥10 cigarettes/day) or weak smoker habit (< 5 cigarettes/day, >5 years of habit). The partially edentulous segment should be in Kennedy’s I or II classification, considering the anterior limit to be on the canine. All patients, where studied before treatment with a panoramic radiography (PR), a computed tomography scan (CT) and a blood test (complete blood count, coagulation tests and glycemia).

- Study Groups

Insertion torque at the time of placement was the variable that determined if the short implants followed a protocol for immediate loading group (ILG) or delayed loading group (DLG). A bridge was immediately loaded when all implants registered an insertion torque of ≥ 40 Ncm. If one or more implants showed a < 30 Ncm resistance, the bridge followed a two-stage procedure (delayed protocol), regardless that other implants supporting the structure could yield the desired values. Bridges with implants inserted with a < 40 Ncm torque but with a resistance of ≥ 30 Ncm were resolved at the surgeon’s criteria. Either a larger diameter implant was used or the implant was left submerged. The insertion torque value was recorded by a drilling unit. The usage of short implants alone (“Shorty bridges” for the purposes of the study) or combined with longer implants (mixed bridges) depended on the length of the opposite arch and bone availability.

- Surgical Procedure

The surgical procedure was performed by a third-grade student (J.A.G) of Master of Oral Surgery and Implantology (Faculty of Dentistry, University of Barcelona, Spain). All implants were placed in healed sites, i.e. at least 3 months after tooth removal allowing the bone to regenerate. Surgery was performed under local anesthesia with articaine 4% and epinephrine 1:100.000 (Artinibsa, Inibsa, Barcelona, Spain); full-thickness mucoperiosteal flaps were raised. The incision was made on top of the alveolar crest and a surgical guide made from the diagnostic wax-up was used to place the implants in the proper place. Brånemark system® Mk III Shorty implants with TiUnite surface (NobelBiocare Göteborg, Sweden), 7 mm in length and 4 mm or 5 mm in diameter, were used alone or in combination with other implants (Brånemark System®MK III TiUnite) of greater length. TiUnite (NobelBiocare Göteborg, Sweden) is a thickened, moderately rough titanium oxide layer with high crystallinity and a high phosphorus content. Drills with increasing diameters were used to under prepare the implant bed depending on the bone quality. The drilling sequence was carried out under profuse irrigation with saline solution or sterile distilled water, with the aim to achieve an adequate insertion torque for immediate load (40 Ncm), measured by a drilling unit, OsseoSetTM 200 (Nobel Biocare). Implants were installed at bone level. Patients were prescribed amoxiciline (Clamoxyl 750, GlaxoSmithKline, Spain; three times a day), and the following analgesics to be taken if required: paracetamol 1g ( Gelocatil, Gelos, Spain;) and ibuprofen 600mg (Espidifen, Zambon, Spain) for 5 days post operatively. Chlorhexidine 0.12% mouthwash (Clorhexidina Lacer, Lacer S.A, Spain) was rec-ommended for 20 days postoperatively.

- Prosthodontic procedure

Prostheses were supported by a maximum of three implants and a minimum of two, depending on the length of the opposing arch and bone availability. Implants that followed the immediate loading protocol were splinted by means of a cemented acrylic resin bridge on Snappy Abutment™ (NobelBiocare) on the day of surgery. The immediate acrylic resin provisional was made from the diagnostic wax-up. After an average provisionalization period of 6 months (range 4-8 months), a definitive metal-ceramic prosthesis was manufactured to provide appropriate oral hygiene and maintenance. All prosthesis were cement-retained.

On the other hand, after a period of 4 months of osseointegration, implants that followed the two-stage protocol were exposed. A mini flap was raised and the Snappy Abutment™ (NobelBiocare) were connected. Finally, the implants were loaded by placing definitive metal-ceramic prosthesis and continuing, from this point on, with the same controls that followed immediately loaded implants.

- Study variables

The variables recorded at the time of surgery were age, gender, whether they were smokers or not, location of edentulous segment, bone quality and quantity, number of implants, implant type and diameter (shorty implants bridge exclusively or shorty associated with longer implants bridge) and insertion torque of each implant placed.

Postoperative follow-up was conducted one week after surgery, coinciding with the removal of sutures and monthly thereafter for those patients following the immediate loading protocol. Data recorded during the follow-up were.

- Probing depth (PD) measured in millimeters using a periodontal probe (PCP 12; Hu-Friedy; Chicago, IL, EE.UU).

- Modified plaque index (mPI) by running the probe across the marginal surface of the implant and recording either the absence or the presence of plaque based on the modified PI (Mobelli *et al*. 1987): no plaque=score 0 o; plaque present=score 1, 2, 3. Positive or negative value (yes/no) was recorded for each implant.

- Modified sulcus bleeding index (mBI) presence or absence of bleeding was recorded by running the probe along the soft tissue margin of the implants. Bleeding was recorded if any site of the implants was positive to the test (score 1, 2, 3 Mombelli *et al*. 1987).

- Suppuration (Sup) presence or absence of suppuration up to 15 seconds after probing was assessed.

- Marginal bone lost at implants was measured by means of periapical radiographs with a positioned (XCP 2000 Instrument, Proclinic, Barcelona, Spain). At the time of the implant placement the first radiographs were obtained (baseline). Bone level change was calculated by subtracting measured bone level at the last follow up visit (June 2012) and measured bone level at surgery day (baseline). Minus (-) was used for values that were below the reference point and plus (+) for values that were above the reference point. Distance was evaluated from the implant-shoulder/prosthesis interface to the marginal bone level in both the mesial and distal sides, while choosing the maximum value as a reference for statistical analysis.

Follow-up visits were carried out by one single examiner (E.D.C). Delayed loading of implants in edentulous areas followed the same protocol once the final prosthesis was fixed.

Survival was defined according to Albrektsson and Zarb’s (9) criteria, regardless the loading protocol followed. Implants with no mobility at the time of evaluation, with no evidence of peri-implant radio lucency or less than 50% of vertical bone loss in the radiography, and with no pain, discomfort or infection associated to the implant, were considered as implants in optimal state. Failed implants were those in one or more clinical-radiological situation previously commented.

- Statistical analysis

All variables were recorded by the same examiner using Microsoft Access for Windows for data collection. A descriptive and bivariate analysis was performed with the Statistical Package for Social Sciences for Windows (SPSS v18.0; SPSS Inc. Chicago, IL, USA). Numerical variables were compared between groups using non parametric U tests Mann-Whitney (two groups) or Kruskal-Wallis (more than two groups). Categorical variables were compared using the contingency table, and dependence is evaluated with the Fisher exact test (equivalent to chi-square test, most appropriate in small sample sizes) or linear association test when ordinal variables can be considered. Marginal bone remodeling and survival rates between different type of bridges and loading protocols, as well as failures associated to smoking habit, were compared by the Chi-square test. Dependence between marginal bone remodeling and initial torque was studied with a correlation test. The significance level was set at p<0.05.

## Results

A total of 26 patients were considered eligible and were consecutively enrolled in this study. Two patients dropped-up the study (one moved out, one did not answer), so they were excluded from the analysis. Accordingly, twenty-four patients (20 women and 4 men), with a mean age of 53.04 years (42-65 range), with 31 partially edentulous free-end areas were analyzed. Shorty bridges were compound of 2 unit (4 immediate and 4 delayed) or 3 unit (4 immediate and 1 delayed) prosthetic bridge, while mixed bridges had 2 unit (4 immediate and 4 delayed) 3 unit (3 immediate and 4 delayed) or 4 unit (2 immediate and 1 delayed) prosthetic bridge ( Table 1).

**Table 1:**
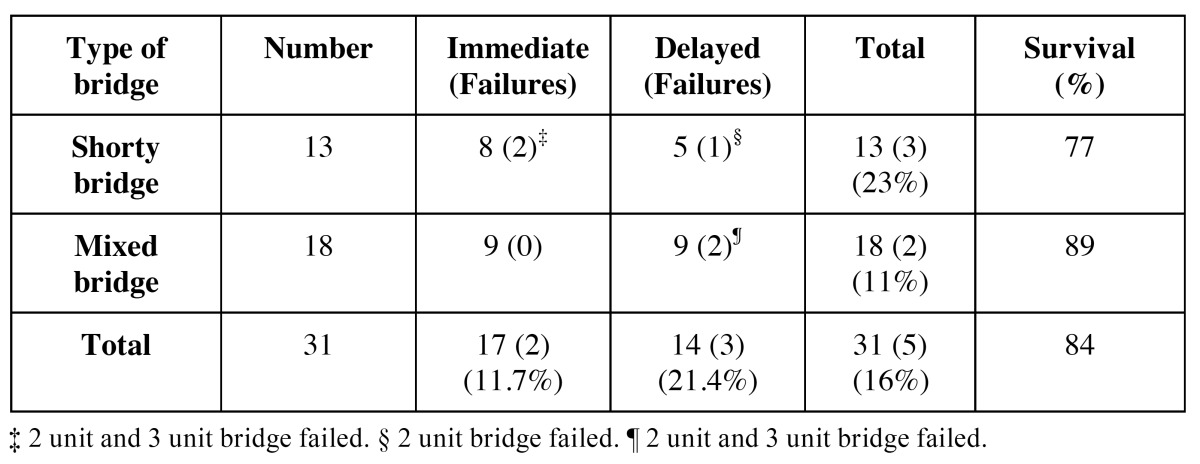
Distribution of free end saddles according to loading protocol and type of bridge.

**Figure 1:**
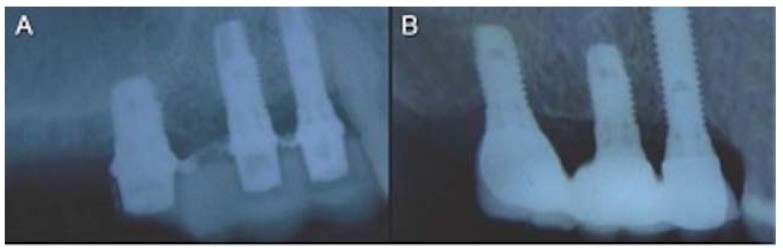
Radiographs of an immediate mixed bridge case. A) Intraoral radiographs of the provisional acrylic resin bridge. B) Intraoral radiographs of definitive mixed bridge after a mean period of 47.72 months of follow-up.

**Figure 2:**
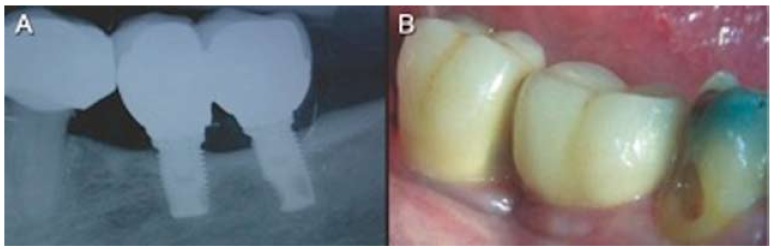
Shorty bridge case. A) Intraoral radiographs of shorty bridge after a mean period of 47.72 months of follow-up. B) Clinical photograph of a shorty bridge.

A total of 69 implants were placed, 54 Brånemark system® Mk III Shorty implants (NobelBiocare) and 15 Brånemark Sys-tem®MK III implants (NobelBiocare) with a length greater than 7 mm, all of them with TiUnite® surfaces (Forty-one implants were placed in the mandible (75,93%) and 13 in the maxilla (24,07%). Of the 54 Shortly implants, 23 were part of bridges supported by different implant lengths (mixed bridges) including, at least, one short implant (Fig. 1); while the remaining 31 were part of bridges supported only by short implants (called “Shorty bridges” for the purposes of the study) (Fig. 2) ( Table 2). These prostheses were supported by a maximum of three implants and a minimum of two, depending on the length of the opposing arch and the available bone. The mean height of the residual bone crest measured in the computed tomography slices was 8.45 mm (range 5-10 mm).

**Table 2:**
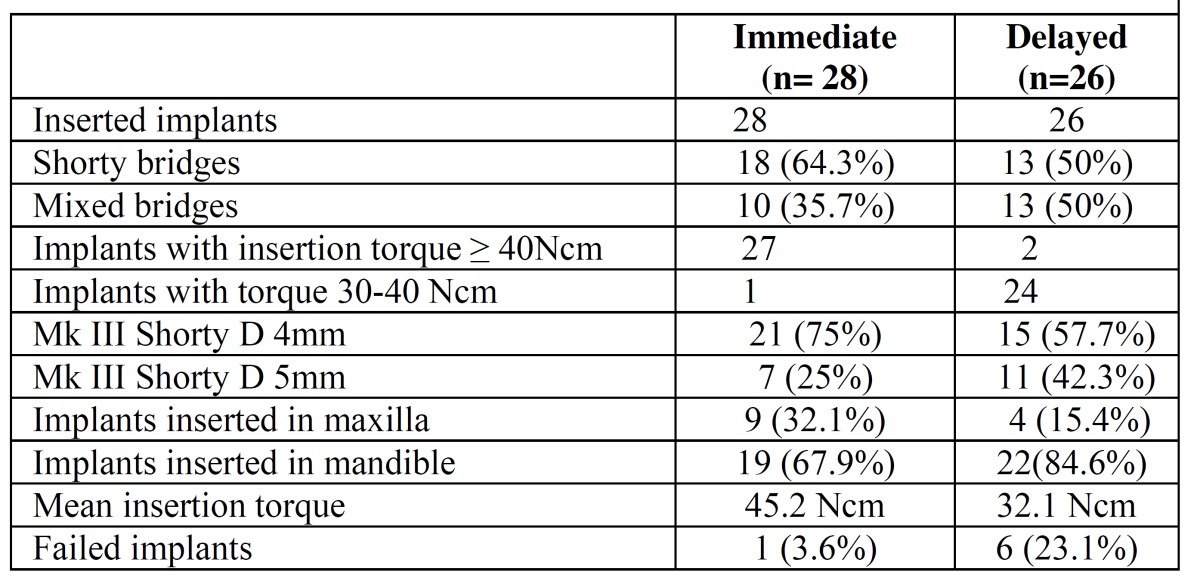
Implant and bridge characteristics.

Insertion torque

The mean insertion torque was 45.2 Ncm (range 40-50 Ncm) and 32.1 Ncm (range 20-35 Ncm) for the 28 immediately loaded and the 26 conventionally loaded short implants, respectively. Four short implants failed to achieve minimum torque values, one implant was immediately loaded, and 3 were conventionally loaded and included in the study.

Survival and implant failures

After a mean follow-up of 47,72 months (range 33-62 months) the cumulative survival rate of short implants was 87% (n=54). However, short implants immediately loaded had a higher long-term survival rate (96.4%) compared to the other study group (76.9%). On the other hand, short implants splinted to longer immediately loaded implants had the higher survival rate (100%). Survival was defined according to Albrektsson and Zarb’s 27 criteria.

Of 54 short implants, a total of 7 implants failed at different time points of the follow-up, showing a statistically significant difference between the location of the implant and implant failure (*p*=0.028). Failure was more common in the maxilla (30.8%) than in the mandible (7.3%). Statistically significant differences were found between loading protocols (*p*=0.047), failure was more frequent in the delayed loading (23.1%) than in the immediate loading protocol (3.6%). Of the 7 implants failed, one was in an immediate shorty bridge, two in a delayed mixed bridge, and four in a delayed shorty bridge ( Table 3). In turn, no implant longer than 7 mm failed during the follow-up period. Four short implants among the failed implants did not achieve the minimum insertion torque required at the first stage.

**Table 3:**
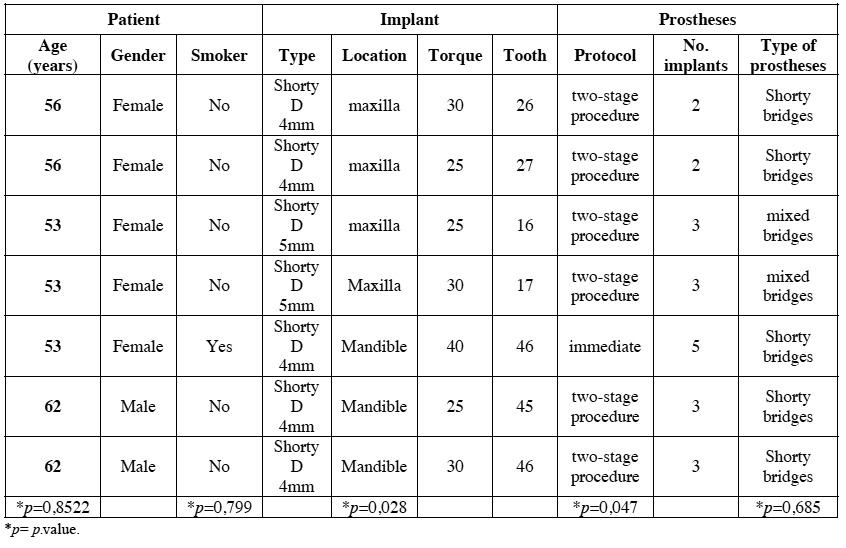
Description of the short dental implants failures.

Marginal bone resorption

**Figure 3:**
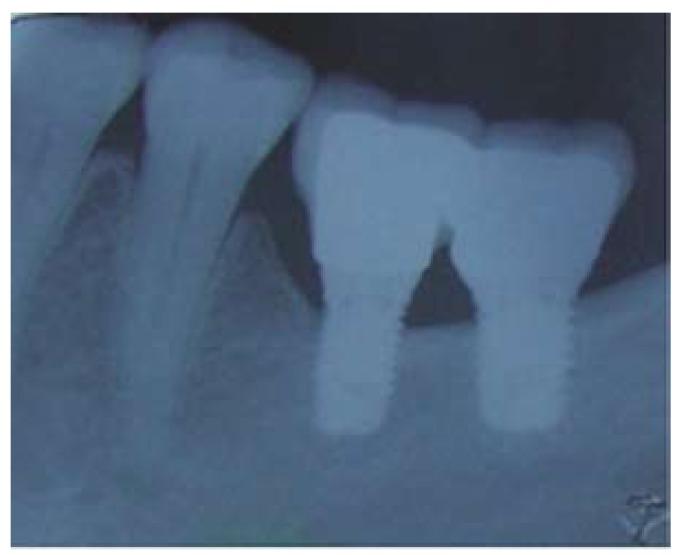
Radiograph 5 years after treatment of two splinted short implants.

Twenty-five (53.19%) short implants showed a bone loss less than one millimeter after a mean period of 47.72 months of follow-up (Fig. 3). Nine short implants (19.15%) showed a marginal bone loss more than two millimeters, while in 13 (27.66%) bone loss was between 1 and 2 mm. There was a slight statistically significant difference between the location of the short implant and bone loss (*p*= 0.066). Implants located in the mandible had a tendency to increased bone loss. There is no statistically significant difference between heavy smoking habit and bone loss (*p*=0.164), although there is a slight tendency to increased bone loss in patients with moderate smoking habit. No statistically significant differences were found either between loading protocols (*p*=0.304) or the bridge type (*p*=0.630). Besides, there were no relation between initial toque insertion and bone loss (*p*=0.208), However, statistically significant differences were found between the presence of plaque, bleeding or suppuration and a higher bone loss (*p*=0.001) ( Table 4).

**Table 4:**
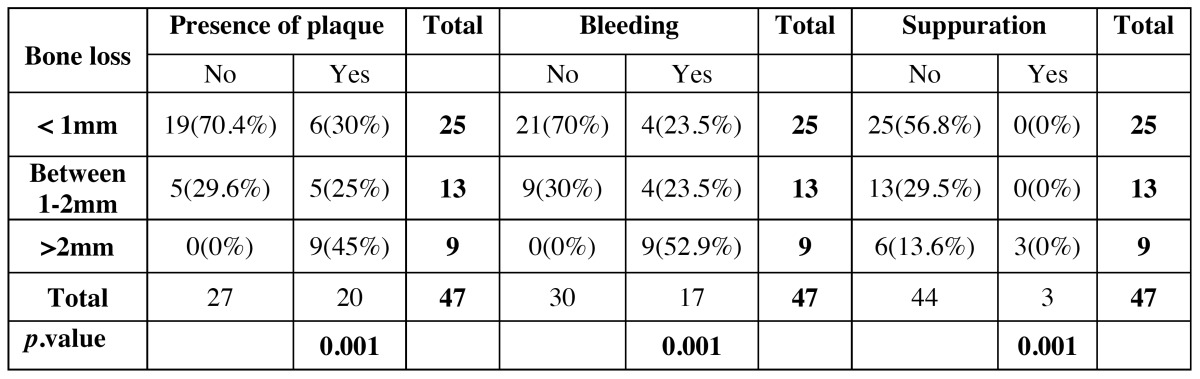
Correlation between marginal bone loss and presence of plaque, bleeding and suppuration.

## Discussion

Bone resorption occurring in partial or total edentulous areas of the maxilla and mandible is one of the main factors that complicate implant planning. The current tendency to reduce morbidity and time spent in a treatment creates a preference for atraumatic techniques (1,2). Short implants are an alternative that requires less traumatic surgery, with fewer complications, dismisses the use of more complex surgical procedures and yields a relatively high survival rate (3,5-8,10-18). However, the use of short implants in the posterior partially edentulous areas may involve a number of disadvantages that include a high crown-implant ratio, excessive functional loading and a less available surface for osseointegration (17-19). If we add to these assumptions the fact that the quality of the bone in posterior areas, especially on the upper jaw, is low, we face several risk factors that may influence negatively the primary implant stability and therefore an immediate loading (17,18).

The purpose of this study was to present preliminary results of immediate loading short implants in posterior areas of partially edentulous jaws, with moderate-severe alveolar bone resorption. The cumulative survival rate of short implants in our study (87.4%) is slightly lower than in other publications, with a range between 88% and 100% (3,7,8). However, if we analyze short implants according to the type of loading, immediate loading registered a 96.4% of survival rates compared with 76.9% of patients whose implants followed a delayed protocol after a mean follow-up of 47.72 months. In a recent retrospective study, Anitua and Orive (10) reported the 1 to 8 years clinical experience with short implants, with a survival rate of 99.3% and 98.8% for the implant and patient base analysis, respectively. These results may clearly demonstrate the predictability and biosafety of the short implants when used under the careful treatment planning and clinical protocol.

Immediate failure of implants may be attributed to several factors, among which are the learning curve, precision drill sequence and quality of bone in the posterior edentulous area. In our study, a last year student of the master degree program in oral surgery and orofacial implantology placed all implants; therefore, this could have influenced the results. Failures at different loading times may be due to functional overload. Some authors propose a set of measures to reduce excessive mechanical load on bone and achieve a better distribution of forces over the prosthetic structure and thus optimize the function of short implants (13,19). This objective is achieved by the following procedures: reduce lateral forces and premature contacts of implant-supported prostheses in partially edentulous ends through proper anterior guidance; avoid the use of “cantilevers” in restorations; splint implants; use implant designs with a larger surface area; and increase the number of implants and their diameter. Wang *et al*. (20) examined the effects that prosthesis materials and splinting have on the stress affecting peri-implant bones under static forces in a finite element model. They established that maximum stress increased, on the basis of the prosthetic material used (resin versus alloy of gold or porcelain), in those implants surrounded by cancelous bone, and recommended to use relatively rigid materials in splinting prosthesis, especially in patients with poor bone quality.

In our study, a total of 7 short implants (13%) were lost at different time points of the follow-up, showing a statistically significant difference respect to its location, and loading protocol. In a recent literature review of 27 studies with implants of ≤8 mm length reported failures between 0 and 14.5%, 0 and 37.5% and 0 and 22.9% of the 6-, 7-, 8-mm long implants, respectively (15). The authors concluded that short implant with machined surface showed generally less favorable results than implants with a rough surface. Implant loss showed a general pattern that concentrated the failures during the healing phase, abutment connection, or at the first year of loading (15). Immediate functional loading is a widely established protocol (21); although there are very well documented clinical data on fixed prosthesis, much better design studies are necessary to provide more scientific evidence (22). Esposito *et al*. (4) concluded that despite having found no statistically differences between implants with conventional or immediate loading, the latter seemed to have a higher failure rate. Furthermore, these authors state that obtaining a high primary stability should be the most important criteria when performing an immediate functional loading, which is a cardinal requirement in our cases. In the present study, statistically differences were found between failures in the different loading protocols, however it is interesting to underline that there were a higher number of failed implants with delayed function, probably due to low insertion torque at the time of placement. It should be emphasized that the low density and poor bone quality may cause failures. In our study the maxilla yielded more failures than the mandible. This finding is in accordance with others reports (15,23). Neldam *et al*. (15), also suggested that is a general tendency to higher failures rates in the maxilla than in the mandible because de poor bone quality. In four of the seven failures observed during the follow-up, probably the low primary stability combined with bone type IV (according to the classification of Lekholm and Zarb) or a lack of primary stability in others were the reasons for these implant failures. Also, short implants are technically demanding and can be associated with short-term failures.

Excessive crown-implant (C/I) ratio (more than 1:1) has been considered in the literature as a decisive long-term prognosis factor in implant survival or could be cause of peri-implant marginal bone loss. However, Birdi *et al*. (24) found no association between crown-implant ratio and the first bone-implant contact in a study of 309 single short implants with fixed restorations. In the same way, Rokni *et al*. (17) in a retrospective study analyzing crown-implant ratio on short implants also concluded that this is not an influencing factor on marginal bone loss, while implant remains stable during loading period in implants between 5 and 7 mm of length. In turn, Pierrisnard *et al*. (16) analyzed stress using a finite element model with different implant lengths (6 to 12 mm in length), but maintaining the same diameter, and conclude that the peak of bone stress is almost constant, regardless of implant length and bicortical anchorage and is not worse for the short ones. Unfortunately, our study did not assess whether the C/I ratio has an impact on marginal bone loss in immediate short implants. This also a default of the present study.

In our study, twenty-five short implants showed a bone loss less than one millimeter, thirteen showed a bone loss between 1 and 2 mm, while in nine the marginal bone loss was greater than two millimeters. No statistically significant differences were found between loading protocols or the type of bridge respect to marginal bone loss. However, statistically significant differences were found between bleeding on probing, presence of plaque or suppuration and a higher bone loss. Peri-implantitis at short implants is an important risk factor for implant failure (25). One mm bone loss around the neck of an implant shorter than 8 mm means a loss of 12.5% bone support. With regard to patients smoking status, there is no significant statistical relationship between the level of smoking and bone loss, although there is a slight tendency to increased bone loss at implants in heavy smoker patients.

Although, there is no uniformity in minimum insertion torque values that may allow high success rates for immediately loaded implants, it seems clear that a high primary stability is fundamental to ensure the survival of implants. In addition to a high insertion torque, the use of rough surface implants and prosthesis splinting are factors that play an important role in the osseointegration processes (7,8,12,17,18,26,27). All these features were taken into account in our study on immediately-loaded of short implants; however, splinted short implants with other of greater length showed a higher long-term survival rate when compared with prosthesis supported by short implants exclusively.

There are a very few studies evaluating clinical outcome of immediate loaded short implants. In a recent study, Cannizzaro *et al*. (28), evaluate the medium-term effectiveness of 6.5 mm long flapless-placed single implants immediately or early loaded. One implant failed in each group giving a success rate of 96.7% in both groups. Implants were inserted with a minimum torque of >40 Ncm. This results are similar in our study if we analyze short implants according to the immediate loading group (96.4%).

The statistical power of the study is low, so it requires a greater number of patients and implants and a longer follow-up. However, we found statistical differences in some of the variables studied. It is important to keep in mind the bias in the group of delayed loading implants as their immediately loading was dismissed on account of the poor bone quality or the lack of surgical precision, which resulted in lower stability and survival rates when compared with other studies of short implants.

## Conclusions

Immediate function of short implants placed on free ends can be considered an option in the treatment protocol of patients with severe bone resorption, especially if implants are splinted to others of greater length. Dental bridges supported only by short implants offer clinically acceptable results in spite of the low morbidity of this technique. One may conclude that, with appropriate case selection, immediate loading of short implants achieves high survival rates, even in cases concerning to bridges supported only by short implants.
